# Late Clinical Outcomes of Total Arterial Revascularization or Multiple Arterial Grafting Compared to Conventional Single Arterial with Saphenous Vein Grafting for Coronary Surgery

**DOI:** 10.3390/jcm12072516

**Published:** 2023-03-27

**Authors:** Justin Ren, Colin Royse, Alistair Royse

**Affiliations:** 1 Department of Surgery, University of Melbourne, Melbourne, VIC 3050, Australia; 2 Department of Cardiothoracic Surgery, Royal Melbourne Hospital, Melbourne, VIC 3050, Australia; 3 Outcomes Research Consortium, Cleveland Clinic, Cleveland, OH 44195, USA

**Keywords:** coronary artery bypass grafting, total arterial revascularization, multiple arterial grafting, radial artery, internal mammary artery

## Abstract

Coronary surgery provides better long-term outcomes than percutaneous coronary intervention. Conventional practice is to use a single arterial conduit supplemented by saphenous vein grafts. The use of multiple arterial revascularization (MAG), or exclusive arterial revascularization (TAR), however, is reported as having improved late survival. Survival is a surrogate for graft failure that may lead to premature death, and improved survival reflects fewer graft failures in the non-conventional strategy groups. The reasons for not using MAG or TAR may be due to perceived technical difficulties, a lack of definitive large-scale randomized evidence, a lack of confidence in arterial conduits, or resources or time constraints. Most people consider radial artery (RA) grafting to be new, with use representing approximately 2–5% worldwide, despite select centers reporting routine use in most patients for decades with improved results. In conclusion, the current body of evidence supports more extensive use of total and multiple arterial revascularization procedures in the absence of contraindications.

## 1. Coronary Revascularization Procedures

The most common cause of death worldwide is coronary artery disease (CAD), which is characterized by the accumulation of atherosclerotic plaques that can rupture and cause thrombosis and myocardial infarction [1]. In severe cases of non-occlusive coronary stenosis, myocardial ischemia may result in heart failure and death [2]. Coronary artery bypass grafting (CABG) and percutaneous coronary intervention (PCI) are two techniques for restoring coronary blood flow [3]. For patients with multi-vessel disease, surgeons must consider lesion complexity, preoperative comorbidities, and other clinical factors. A number of randomized studies (RCT) comparing CABG against PCI have demonstrated improved outcomes of CABG in patients with diabetes and high disease burden [4,5,6] without evidence for higher periprocedural mortality. Compared to PCI, CABG significantly improved long-term outcomes [7,8,9] likely due to a new blood supply route that is less affected by diffuse disease [10]. In the 5-year SYNTAX trial analysis, Kaplan–Meier estimates of myocardial infraction (CABG, 3.8%; PCI, 9.7%; *p* < 0.0001), repeat revascularisation (CABG, 13.7%; PCI, 25.9%; *p* < 0.0001), and major adverse cardiac and cerebrovascular events (CABG, 26.9%; PCI, 37.3%; *p* < 0.0001) were significantly higher in the PCI group compared to the CABG group [8]. Similarly, an individual patient meta-analysis of 11 RCTs [9] identified a 12% increase in 5-year mortality risk after PCI (hazard ratio [HR], 1.2; 95% confidence interval [CI], 1.06–1.37; *p* = 0.004). In a Bayesian analysis, there was an 85.7% probability that PCI had greater risk of death at 5 years than CABG [11].

## 2. Total Arterial Revascularization

About 95% of global CABG procedures currently involve the use of left internal mammary artery (LIMA) and saphenous vein grafts (SVG) (LIMA + SVG) [12]. Because of progressive failure of SVG related to accelerated atherosclerosis [13,14], alternative revascularization techniques without SVG are being sought. Reduced use of SVG by using greater numbers of arterial grafts is referred to as multiple arterial grafting (MAG), and exclusive use of arterial conduits without any SVG is referred to as total arterial revascularization (TAR). The surgeon seeks to minimize the risk of heart failure or death [15] associated with SVG’s known progressive failure in this way. Atherosclerosis does not progress in arterial grafts, which results in improved long-term durability and, in turn, protects native vessels against atherosclerosis [16]. In a meta-analysis of 5 RCTs with a median follow-up of 10 years [17], SVG use was associated with higher mortality (HR, 0.73; 95% CI, 0.57–0.93; *p* = 0.010) than radial artery (RA), consistent with other meta-analyses [18,19]. Data from decades of studies support the use of the internal mammary artery (IMA) over the saphenous vein graft (SVG) [20,21,22]. A study by Cameron et al. assessed the long-term results (16.8 years) of 5637 patients, documenting the use of IMA as an independent predictor for survival in multivariable analysis. The survival advantage was consistently observed regardless of sex, age, ventricular function, and left main disease [21].

**Figure 1:**
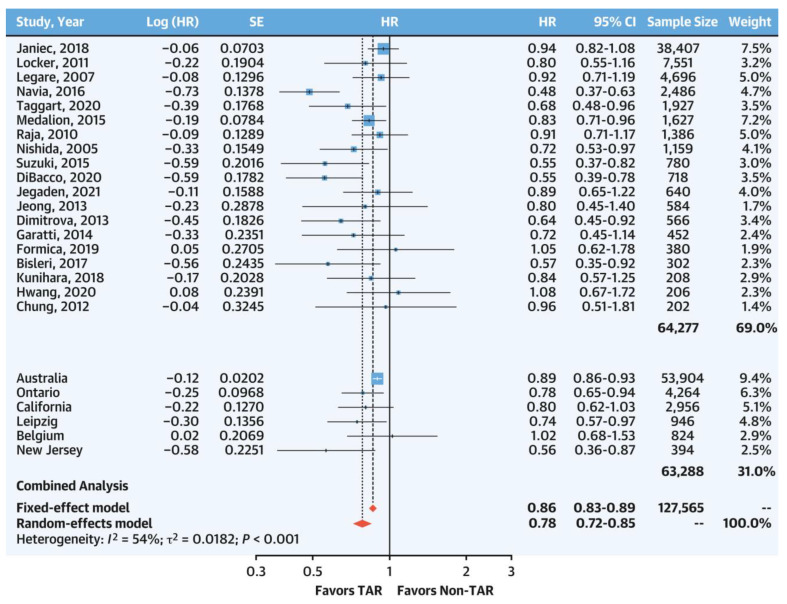
Total arterial revascularization vs. non-total arterial revascularization: combined literature review and expanded multicenter collaborative data set meta-analysis. Reproduced with permission [23] Royse A, et al., J Am Coll Cardiol. 2022; 80(19):1833–1843. Data from individual publications or registries were pooled using a meta-analysis methodology. A total of 4 studies from the literature review section were removed because they included, or potentially included, duplicate patients from the expanded multicenter collaborative data set. A combined cohort of 127,565 propensity-score-matched or propensity-score-adjusted patients allocated to total arterial revascularization or non-total arterial revascularization were compared for late all-cause mortality at 8.3 years (95% CI: 6.2–10.4 years). An HR < 1 indicates a survival benefit. Almost all studies found a survival benefit favoring total arterial revascularization. Meta-analysis for the combined cohort found a significant survival advantage for total arterial revascularization by both random-effect and fixed-effect models.

**Figure 2:**
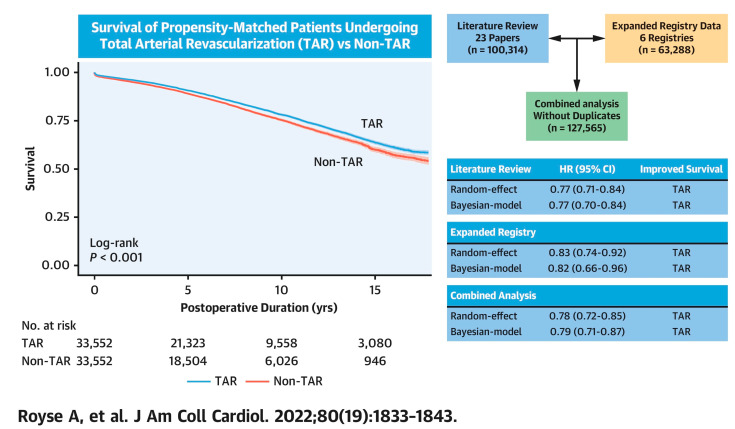
Survival advantage for total arterial revascularization in coronary bypass grafting. Reproduced with permission [23] Royse A, et al., J Am Coll Cardiol. 2022; 80(19): 1833–1843. From a combination of literature and expanded current international registry data in matched patients. Survival for those exclusively receiving arterial coronary grafts (TAR) was greater than those that received supplementary saphenous vein grafts (non-TAR).

Based on a systematic review of the current literature and multi-national collaborative registry data (n = 127,565 adjusted patient pairs), our group compared patients receiving TAR to those not receiving TAR. We found that the complete avoidance of SVG or TAR reduced long-term all-cause mortality (HR, 0.78; 95% CI, 0.72–0.85; *p* = 0.010) after propensity score and Cox-regression adjustments (Figure 1). This was confirmed by a Bayesian analysis suggesting a > 99.9% posterior probability that SVG was the cause of the lower observed late survival in the non-TAR group and a 99.8% probability for an effect size of HR < 0.9 [23]. Kaplan–Meier survival analysis on the digitized individual patient data indicated an early and incremental separation between TAR and non-TAR over time (Figure 2) (log rank *p* < 0.001). Our results were consistent with previous meta-analyses [24,25]. SVG has a well-known atherosclerotic behavior and high rate of late graft failure. It could then be reasonably postulated that the use of any SVG may likely be the mechanism by which long-term survival is diminished.

The saphenous vein graft contributes to almost 80% of international CABG practice [26], often prepared by adventitia dissection and manual distension during conventional harvesting. The “no-touch” harvesting technique was proposed as an attempt to enhance the long-term patency of SVG and thus patient survival, which has been one of the most crucial challenges associated with SVG usage. It has been suggested that surgeons could harvest the vein with a pedicle of surrounding tissue to reduce vascular damage and preserve structural integrity [27]. While a large multicenter randomized trial (2655 patients) found a 40% relative increase in SVG patency 12 months after no-touch harvesting compared to the conventional pedicled fashion, no clinical benefit was observed, including survival [28]. Another multicenter trial by Deb and colleagues, however, observed no patency improvement at 12-month follow-up [29]. Therefore, it remains unknown whether this technique would confer any meaningful benefits.

To date, few randomized trials have compared TAR with conventional procedures. Muneretto et al. [30] randomized 200 patients over 70 years of age to either LIMA + SVG or composite (Y-graft) TAR. Composite is a term to describe the joining of conduits together allowing one or more conduits not to be anastomosed to the ascending aorta. As a result, the same length of conduit could reach more target coronary arteries or reduce aortic manipulation by eliminating aortic anastomosis where atheroma may exist. The conventional LIMA + SVG cohort was identified as a risk factor for recurrent angina and graft occlusions. There were no significant differences in graft patency, in-hospital mortality, or stroke between TAR and non-TAR cohorts in a trial led by Le and colleagues [31] of 58 primary isolated CABG patients at 6 months post-operatoin likely due to a short follow-up duration and a small sample size.

In many countries, particularly in North American regions, adoption rates of TAR are very low (10%) [32] and resistance among surgeons to the use of TAR may relate to the following: (1) perceived increased technical difficulties associated with arterial harvesting and revascularization, (2) perception of increased postoperative complications such as sternal wound infections where bilateral internal mammary arteries are used, (3) longer operation duration due to unfamiliarity with the techniques, (4) absence of a large-scale prospective randomized trial, and (5) lack of financial incentives for the surgeons in the context of perceived greater duration of operations and greater complexity. Furthermore, TAR is commonly perceived to require more preoperative assessment and to be unsuitable for a large proportion of patients. It is believed by those who practice routine TAR that these attitudinal or psychological barriers are not accurate perceptions and may be related to a lack of familiarity or experience with these techniques, leading to an intrinsically biased practice within the surgical community.

We consider achieving TAR to be relatively straightforward for most patients by any surgeon currently performing CABG. A majority of CABG series report a mean graft use of 3.0–3.3 grafts per patient [32,33,34,35]. In the case of surgeons’ usual grafting technique, simple substitution of an arterial conduit for SVG will significantly increase MAG and TAR. For example, one LIMA and two RA would allow for three grafts, and one sequential graft or alternatively a second internal mammary artery would allow for four grafts. In this way, more than 80% of CABG cases would be able to rely solely on arterial conduits with conventional grafting techniques, thereby addressing the majority of technical issues.

## 3. Bilateral Internal Mammary Artery Grafting

Consistent evidence on the long-term benefits of using LIMA in CABG has stimulated widespread interest from surgeons in the use of the supplementary right internal mammary arteries (RIMAs) due to their biological homogeneity [22]. The first myocardial revascularization incorporating bilateral internal mammary arteries (BIMAs) was described by Suzuki and colleagues in the treatment of diffuse coronary artery disease. Several meta-analyses and observational studies [36,37,38,39] have documented BIMA’s benefits for TAR. A network meta-analysis of 35 studies with a mean follow-up duration of 6.9 years showed that BIMA was associated with improved survival [36]. The arterial revascularization trial (ART) was the largest multi-center unblinded RCT which enrolled 3102 patients assigned to receiving either bilateral or single internal mammary artery grafting [40]. The as-treated analysis in the BIMA cohort confirmed a survival advantage, but the intention-to-treat analysis, for which randomization was designed, found no difference in all-cause mortality after 10 years. This may be due to a high crossover rate (16.4%) and the frequent use of the radial artery (23.1%), which was considered at the time of study design to be equivalent to SVG rather than LIMA. Both arms of the study also received supplementary SVG, and this may have influenced the outcome as was evident in a post hoc analysis of TAR versus non-TAR, which found a significant survival advantage for TAR [41]. 

Despite compelling evidence and guidelines [42,43], BIMA remains underutilized in CABG worldwide with 4.1% in the US [44], 12.6% in Australia [45], 12% in Europe, and 34.9% in Japan. BIMA has been vigorously debated due to concerns about deep sternal wound infections (DSWI), especially in diabetics and other high-risk groups. Gaudino et al. [36] reported that the use of RIMA as a second conduit had a higher risk of DSWI than both SVG (odds ratio [OR], 1.41; 95% CI, 1.10–18.2) and RA (OR, 1.39; 95% CI, 0.92–2.1). The relationship between differential harvesting techniques and sternal wound infections remains controversial. A pedicle technique harvests IMA together with the endothoracic fascia, adipose tissues, and lymphatics, while skeletonization requires IMA to be dissected free of all surrounding tissues. Studies consistently demonstrated that skeletonization would reduce the damage to retrosternal microcirculation, maintain greater residual blood perfusion, and enhance local oxygen saturation compared to the conventional pedicle technique [46,47]. Another sternal vascularity study using single photon emission computed tomography observed decreased blood flow only when ITA was harvested in a pedicled fashion [48]. From a clinical aspect, the results of a meta-analysis [49] involving 2633 patients confirmed a significantly improved freedom from DSWI after skeletonized ITA harvesting (OR, 0.327; 95% CI, 0.217–0.492; *p* < 0.001). 

## 4. Radial Artery Grafting

The first RA grafting procedure was documented by Carpentier and colleagues [50] but was soon abandoned due to higher occlusion rates than SVGs, though this experience was often not published. It was thought that the endothelial lesions from mechanical dilation and skeletonized harvesting might have led to vascular spasm and intimal hyperplasia [51,52], thus causing graft failures. The use of RA as a bypass graft was resurrected in 1992 where Acar et al. reported a series of 102 patients [53]. The major differences between the two studies were claimed to relate to technical modifications that reduce endothelial damage, including the replacement of mechanical dilation with pharmacological dilation and pedicled harvesting instead of skeletonized harvesting. To date, there remains a paucity of evidence to support these hypotheses.

For the second most important coronary target with severe stenosis, the 2021 AHA guideline now recommends RA instead of both SVG and RIMA. An analysis of 14 adjusted observational series with a mean follow-up duration of 6.6 years found that the RA group had a 26% relative reduction in mortality risk compared to the SVG group. As a result of a pooled individual patient-level analysis of 6 RCTs, RA was associated with fewer graft occlusions (HR, 0.44; 95% CI, 0.28–0.70), with a lower risk of adverse cardiac events at 5 years (HR, 0.67; 95% CI, 0.49–0.90) and 10 years (HR, 0.73; 95% CI, 0.61–0.88) postoperatively [17,54]. The recent 15-year RAPCO extension study confirmed a significantly lower rate of MACE associated with RA grafting in comparison to SVG [55]. Alternatively, patients undergoing BIMA are at a higher risk of sternal wound infection, which would make RA a safer option during preoperative planning [56]. Surgeons have reported other technical advantages in the use of RA, including reduced operation time since it can be harvested at the same time as LIMA, adaptable length to reach distal targets, and easy handling due to thick muscular layer [57,58].

**Figure 3:**
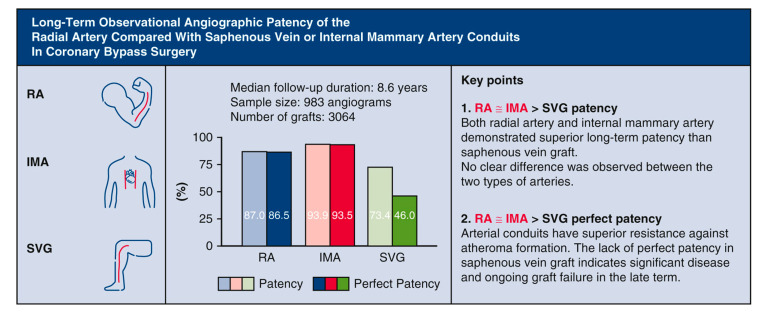
A summary of our retrospective study. Reproduced with permission [34] Ren, J. Long-term observational angiographic patency and perfect patency of radial artery compared with saphenous vein or internal mammary artery in coronary bypass surgery, J Thoracic Cardiovasc Surg 2022, 10.1016/j.jtcvs.2022.08.047. Abbreviations: OR, odds ratio; RA, radial artery; IMA, internal mammary artery; SVG, saphenous vein graft; CABG, coronary artery bypass grafting; Cx, circumflex artery; IMA, internal mammary artery; IQR, interquartile range; LAD, left anterior descending artery; OR, odds ratio; PCI, percutaneous coronary intervention.

Although there is a significant amount of evidence suggesting that RA is superior to SVG, few studies have directly compared RA with IMA. From 1997 to 2020, our group examined the long-term angiographic patency of three major conduit options [34]. The multivariable adjusted analysis of 3064 grafts found a higher graft patency rate of RA vs. SVG (OR, 3.37; 95% CI, 2.23–5.08; *p* < 0.001) and IMA vs. SVG (OR, 4.72; 95% CI, 2.74–8.15; *p* < 0.001) (Figure 3, Table 1), with even greater difference for perfect patency indicative of atherosclerotic disease. However, we reported no difference in patency or perfect patency between 2 arterial grafts, suggesting that RA has a similar conduit behavior as IMA (patency OR, 1.40; patency 95% CI, 0.85–2.33; patency *p* = 0.189; perfect patency OR, 1.14; perfect patency 95% CI, 0.71–1.84; perfect patency *p* = 0.578) with no ongoing atheroma formation. Although the absolute patency was higher in IMA (93.9%) compared with RA (86.9%), the difference may relate to target coronary territory where IMAs were preferentially grafted to the left anterior descending (LAD) territory with the largest blood flow. The discrepancy was no longer significant after systematic multivariable adjustment. Our analysis later confirmed that LAD territory was associated with higher patency outcomes than either circumflex or right coronary artery territory irrespective of conduit type. 

In our subset analysis by follow-up duration, early patency reduction appeared between 0 and 6 months, potentially relating to the residual coronary blood flow in line with expectations for arterial conduits. However, this was unexpected for SVG which was believed to have natural resistance against native flow competition.

**Figure 4:**
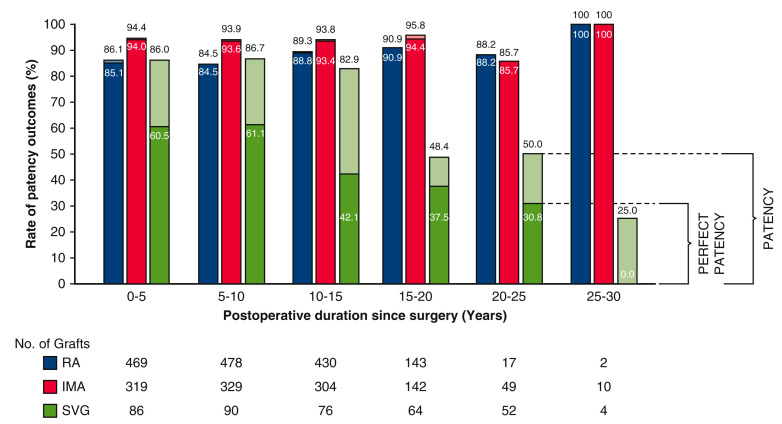
Rate of patency and perfect patency of unique individual grafts. Reproduced with permission [34] Ren, J. Long-term observational angiographic patency and perfect patency of radial artery compared with saphenous vein or internal mammary artery in coronary bypass surgery, J Thoracic Cardiovasc Surg 2022, 10.1016/j.jtcvs.2022.08.047. Lighter colors represent patency, and darker colors represent perfect patency. All observations are directly measured without statistical estimation. For example, the bar of 5 to 10 years postoperative captures all angiograms taken within this time frame. Additional analysis up to 1 year postoperative shows that reductions in patency are evident within 3 months of surgery and that patency and perfect patency remains similar in later periods. Abbreviations: RA, radial artery; IMA, internal mammary artery; SVG, saphenous vein graft.

The long-term resistance against conduit atherosclerosis was shown by all patent arterial conduits exhibiting a normal angiographic lumen, indicating an absence of conduit wall atherosclerosis. The same finding was observed at all time points, even after more than 20 years. Perfect patency for SVG was lower than for either IMA or RA at all postoperative time points (Figure 4), indicating ongoing development of atherosclerosis and progressive failure consistent with the RAPCO trial [33].

The RAPCO trial was the only long-term angiographic randomised comparison of RA patency to both free RIMA and SVG to our knowledge, though this was not a serial angiographic study of the same patients or conduits over time. The authors found that RA harvesting was not associated with increased risk of sternal wound infections as distinct from BIMA procedures reported by other meta-analysis [59]. In a quality-of-life survey [60] conducted by RAPCO investigators, about 8% of patients reported pain and numbness in hands and forearms after RA procurement that peaked at 3-month after surgery and diminished during long-term follow-up. It was suggested that functionality of hands and forearms declined during long-term follow-up, but it was likely related to age rather than harvesting. Most importantly, RA harvesting was associated with higher patient satisfaction and less scar formation compared to SVG harvesting. Another series of 211 patients undergoing neurological assessment of the hand at a mean follow-up duration of 26 months documented a 10–15% prevalence of numbness [61], which was much higher than RAPCO. The experience level of surgeons and the institution volumes of RA procurement are deterministic factors to the rate of postoperative neurological complications. Another prospective study of the Veterans Affairs database observed slightly greater pain in RA harvesting than SVG harvesting, but the pain was not severe and often solved within 12 months postoperatively. Grip strength, manual dexterity, and neurological functions of the harvesting site were similar among both cohorts [62].

The safety of radial artery harvest is high, and functional outcomes for the hand and forearm are minimal [63]. In addition to the conventional Allen’s test, using ultrasound as a confirmatory test can also provide information on vascular diameter and plaque formation [64]. Based on theoretical considerations, many believe RA is more susceptible to vasospasm than SVG due to its predominantly muscular wall [65]. Calcium-channel blockers (CCBs) have traditionally been prescribed for all patients with RA grafts [66], but other authors have found indifferent clinical and angiographic outcomes after taking CCBs. [67,68]. Our group practice with tens of thousands of RA have observed RA spasm infrequently and without any clear correlation with the use of vasoconstrictors which have been used frequently or routinely.

Reduced RA patency has been observed in the presence of native coronary flow through a mild or moderate stenosis that then competes with the graft. The mechanism by which an arterial graft may fail in these circumstances has not been fully elucidated but may result from a reduced supply of nutrition to the conduit. The analyses of Cleveland Clinic [69] and Westmead Hospital [70] of symptom-driven angiographic data have demonstrated a linear relationship between IMA graft occlusion and native coronary stenosis, which is consistent with our finding of the effect of preoperative stenosis on patency outcomes [34]. The fractional flow reserve (FFR) provides an improved preoperative assessment of coronary stenosis severity than conventional visual inspections. Limited and inconsistent evidence exists over the recommended threshold for severe stenosis, but many studies have suggested grafting RA to coronary targets with >70% stenosis [71,72,73]. Based on our retrospective review of RA grafts from the mid-1990s, patency rates were higher for RA grafted to coronary stenosis 50–70% than generally reported in the literature [74]. A relative contraindication to the use of RA conduit would be trans-radial catheterization procedures which may cause morphological and functional impairments, including reduced vessel diameters, vasomotor functions, and structural damages to the wall [75]. Our practice now commonly uses RA that have been used for angiography, even within days of the procedure, after an ultrasound examination has excluded significant vascular injury. In addition, it is common to not need the entire length of the RA to reach a specific target coronary artery, so the direct puncture site may not be included in the conduit.

## 5. Multi-Arterial Grafting

A large body of evidence has uniformly suggested that MAG is associated with superior clinical outcomes to SAG. A multi-center retrospective study of 8629 patient pairs reported that MAG had significantly lower risk of mortality (HR, 0.80; 95% CI, 0.73–0.88; *p* < 0.01) and MACCE (HR, 0.92; 95% CI, 0.77–0.88; *p* < 0.01) at 8 years after procedures compared to SAG [76]. Similarly, another 10-year propensity-adjusted study conducted by Chikwe et al. [77] showed that multi-arterial CABG was associated with lower mortality (HR, 0.84; 95% CI, 9.76–0.92; *p* < 0.001) than single-arterial CABG. 

Despite being recommended by official guidelines for patients with reasonable life expectancies, multiple arterial grafting (MAG) with more than one arterial graft remains rarely used by surgeons [3]. In contrast to TAR, MAG does not eliminate the use of SVG, but instead increases the number of arterial conduits. The only comparison between MAG and TAR was from a large meta-analysis conducted by Yanagawa et al. investigating clinical outcomes, where the authors identified a trend of better survival in the TAR cohort (HR, 0.80; 95% CI, 0.62–1.05; *p* = 0.11). There were no differences in short-term myocardial infarction, stroke, or mortality [25]. In addition, an exploratory post hoc analysis by the ART investigators showed an incremental benefit from SAG to MAG and then TAR for 10-year mortality and a composite of death, myocardial infarction, stroke, and repeat revascularization, suggesting that TAR may provide the greatest benefit [41]. There will be greater clarity after the ROMA trial is completed [78], which could become a piece of deterministic prospective evidence comparing multiple against single arterial grafting across international centers. 

The application of MAG is largely reduced in women [79] due to concerns with a higher prevalence of cardiovascular risk factors and worse clinical prognosis compared to men [80,81]. However, sex-differentiated analyses of MAG vs. SAG consistently favored the use of more arterial conduits. At a median follow-up duration of 5 years, Tam et al. [82] presented matched results in females that MAG improved survival (HR, 0.85; 95% CI, 0.74–0.98) and freedom from MACCE (HR, 0.85; 95% CI, 0.76–0.95) compared to SAG. After competing risk adjustment of death, lower incidence of repeated revascularization (HR, 0.77; 95% CI, 0.64–0.93) was also observed in the multi-arterial grafted female cohort. Similarly, another observational study from New York’s Cardiac Surgery Reporting System [83] confirmed a significant association of MAG, with longer survival in both men and women although there was a risk-threshold for such benefit. It is possible, however, that the retrospective study design had unmeasured confounders and selection bias despite extensive statistical adjustment. A change of practice would therefore require more definitive sex-differentiated evidence, but female patients remain underrepresented in contemporary randomised clinical trials on revascularization techniques.

## 6. Conclusions

Total arterial revascularization and multiple arterial grafting could be achieved by RA and IMA, and both have superior angiographic and clinical outcomes compared to the conventional single-arterial strategy. Major barriers against their wider application in international practice relate to perception of increased technical difficulties, increased postoperative complications, longer operation duration, absence of a large-scale prospective randomized trial, and lack of financial incentives for the surgeons. IMA and RA have similar patency, while both have superior patency to SVG. BIMA is associated with increased risk of deep sternal wound infection, but skeletonized BIMA harvesting may preserve tissue integrity and thus minimize postoperative sternal infections in contrast to the conventional pedicled technique. RA grafting has numerous technical advantages, including reduced operation time, adaptable length to reach any target, and simple handling. Trans-radial catheterization could be a contraindication for RA grafting. Limited investigations compared TAR against MAG and suggested better long-term outcomes after TAR, but further large-scale trials are required to confirm this finding. In conclusion, the current body of evidence supports more extensive use of total and multiple arterial revascularization in coronary bypass procedures.

## Figures and Tables

**Table 1:** Comparison of graft patency and perfect patency according to individual anastomoses at 8.6 years postoperatively.

Comparative Analysis	Patency		Perfect Patency	
	Odds Ratio (CI)	*p*	Odds Ratio (CI)	*p*
***Overall effect of conduit type***	-	<0.001	-	<0.001
**RA vs. SVG**	3.37 (2.23, 5.08)	<0.001	17.57 (11.39, 27.08)	<0.001
**IMA vs. SVG**	4.72 (2.74, 8.15)	<0.001	20.11 (11.64, 34.74)	<0.001
**IMA vs. RA**	1.40 (0.85, 2.33)	0.189	1.14 (0.71, 1.84)	0.578

Reproduced with permission [34] Ren, J. Long-term observational angiographic patency and perfect patency of radial artery compared with saphenous vein or internal mammary artery in coronary bypass surgery, J Thoracic Cardiovasc Surg 2022, 10.1016/j.jtcvs.2022.08.047. Multivariable analysis of 3064 grafts from 983 patients; adjusted except for preoperative stenosis. Abbreviations: RA, radial artery; SVG, saphenous vein graft; IMA, internal mammary artery; CI, 95% confidence interval.

## Data Availability

Data sharing not applicable since no new data was generated.
